# Decoration of MnFe_2_O_4_ nanoparticles on activated carbon as recoverable photocatalyst for perfluorooctanesulfonic acid degradation in water

**DOI:** 10.1039/d6ra00405a

**Published:** 2026-03-25

**Authors:** Nguyen Trung Kien, Le Bao Hung, Nguyen Quang Bac, Nguyen Thi Ha Chi, Pham Ngoc Chuc, Do Nguyen Huy Tuan, Nguyen Tran Dung, Truong Minh Tri, Nguyen Vu Ngoc Mai, Dao Ngoc Nhiem

**Affiliations:** a Institute of Materials Science, Vietnam Academy of Science and Technology 18 Hoang Quoc Viet Street, Nghia Do Ward Hanoi 100000 Vietnam nhiemdn@ims.vast.ac.vn; b Graduate University of Science and Technology 18 Hoang Quoc Viet Street, Nghia Do Ward Hanoi 100000 Vietnam; c Joint Vietnam-Russia Tropical Science and Technology Research Center 63 Nguyen Van Huyen Street, Nghia Do Ward Hanoi 100000 Vietnam; d Mientrung University of Civil Engineering 195 Ha Huy Tap Street, Binh Kien Ward Dak Lak 630000 Vietnam; e Faculty of Natural Science, Quy Nhon University 170 An Duong Vuong Street, Quy Nhon Nam Ward Gia Lai 610000 Vietnam nguyenvungocmai@qnu.edu.vn

## Abstract

A MnFe_2_O_4_/activated carbon composite (FMAC) was successfully prepared for the treatment of perfluorooctanesulfonic acid (PFOS). Structural and compositional analyses confirmed the formation of spinel manganese ferrite (FMO) nanoparticles and their uniform immobilization on the activated carbon (AC) surface. XPS results revealed the coexistence of multiple oxidation states of Mn and Fe, along with abundant oxygen-containing functional groups on the carbon support, indicating strong interfacial interactions between FMO and the AC support. Optical measurements showed that FMO and FMAC exhibit excellent visible-light absorption with a bandgap (*E*_g_) of ∼2.4 eV. Electrochemical studies also demonstrated reduced charge transfer resistance and suppressed electron–hole recombination in FMAC. Vibrating sample magnetometry (VSM) also indicated a sufficient magnetic response for recovery of FMAC from aqueous media. Photocatalytic tests showed that FMAC with a content of 10 mg L^−1^ accomplished significant degradation efficiency (∼94%) of PFOS in 100 ppb solution at pH 4.0 under visible light irradiation for 90 min, which was higher than both FMO and AC under similar conditions. Radical scavenging experiments indicated that holes and hydroxyl radicals played dominant roles, while superoxide radicals were negligible due to unfavorable band edge potentials. Inclusively, the enhanced performance of FMAC is attributed to improved pollutant adsorption, efficient charge separation, and strong interfacial coupling between FMO and AC.

## Abstract 2

A MnFe_2_O_4_/activated carbon composite (FMAC) was successfully prepared for the treatment of perfluorooctanesulfonic acid (PFOS).

## Graphical abstract

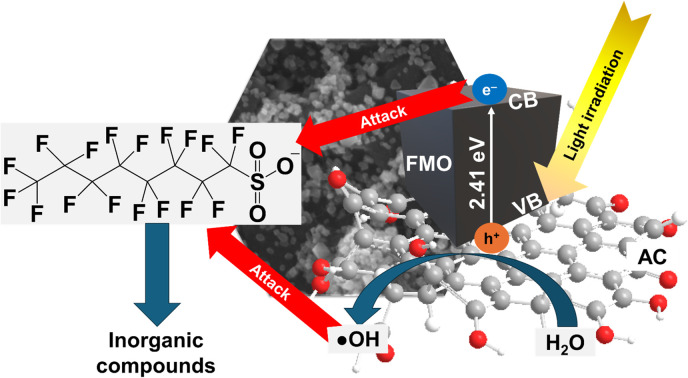

## 1. Introduction

Water quality degradation has become a global concern driven by population growth, rapid urbanization, and expanding economic activities. Conventional wastewater treatment plants (WWTPs) are often ineffective in removing organic micropollutants, allowing these contaminants to enter surface and groundwater systems.^1^ Consequently, numerous emerging and persistent pollutants are now widely detected in aquatic environments. Among them, perfluorinated compounds (PFCs), characterized by a perfluoroalkyl chain –CF_3_–(CF_2_)_*n*_– attached to hydrophilic functional groups (*e.g.*, –COOH, –SO_3_H, –SO_3_NH–, or –CONH_2_), have attracted significant attention due to their exceptional resistance to hydrolysis, photolysis, and biodegradation.^2,3^ Owing to their unique surface-active and thermal stability properties, PFCs have been extensively used in industrial and consumer applications, including textile coatings, polymer production, firefighting foams, paper treatment, lubricants, and pesticides.^4^ Perfluorooctane sulfonate (PFOS) is one of the most representative and problematic PFCs. It exhibits high persistence and long environmental and biological half-lives (*e.g.*, ∼4.8 years in humans and up to decades in aquatic systems).^5–7^ In 2009, PFOS was listed as a persistent organic pollutant (POP) under the Stockholm Convention.^8^ Despite global restrictions, PFOS remains detectable in surface water, groundwater, and drinking water due to its historical usage and chemical stability.^9,10^ Monitoring studies across major oceans have identified PFOS as a dominant PFC, accounting for approximately one-third of total detected PFCs worldwide.^2^ The persistence, bioaccumulation potential, endocrine-disrupting effects, and toxicity of PFOS and related PFAS underscore the urgent need for effective and economically viable treatment technologies.

Traditional treatment methods, including adsorption, coagulation, membrane filtration, and biological processes, often show limited effectiveness for PFOS removal or merely transfer the contaminant from the aqueous phase to another medium.^11–14^ To address the challenge, advanced oxidation processes (AOPs) have emerged as promising alternatives for the destructive treatment of diverse hazardous contaminants, including POPs like PFCs and PFOS. AOPs depend on the *in situ* formation of greatly reactive species, particularly hydroxyl radicals (˙OH), capable of breaking strong chemical bonds and promoting partial or complete mineralization of organic pollutants. Despite this potential, several studies have reported negligible PFOS degradation when using common AOPs such as ozonation or Fenton processes, even after prolonged treatment times, which was mainly attributed to the exceptional stability of the C–F bond.^15^ On the other side, heterogeneous photocatalysis, a subclass of AOPs, has placed itself as a potentially effective approach for destruction and mineralization of PFOS. This process involves the activation of a semiconductor photocatalyst under UV or visible light, leading to the generation of electron–hole pairs and reactive oxygen species that can oxidize organic pollutants. Although TiO_2_ has been the most extensively researched photocatalyst due to its durability, cost effectiveness, and environmentally friendliness, its broad bandgap (∼3.2 eV) limits its activity mainly to ultraviolet light, which constitutes a minor portion (<5%) of the solar spectrum.^16^

Recently, magnetic iron oxide-based materials and ferrites with the general formula MFe_2_O_4_ (M = Mn, Co, Ni, Zn, *etc.*) have gained attention as alternative visible-light-active photocatalysts for treatment of various contaminants, including POPs.^17–19^ MFe_2_O_4_-based materials are semiconductors with narrow bandgaps enabling efficient absorption of visible light.^20^ In addition, MFe_2_O_4_ also exhibits strong magnetic nature, facilitating catalyst recovery and reuse, as well as limiting the generation of secondary pollutants, which are the important advantages for water treatment applications.^20,21^ However, MFe_2_O_4_-based photocatalysts may still suffer from their disadvantages such as particle agglomeration, low surface area, and fast photogenerated charge recombination.^19,21^ To address these issues, mixed-metal ferrites and composite materials have been developed.^22^

Carbonaceous materials, including AC, biochar, graphene, and carbon nanotubes, have been extensively utilized for the removal of organic pollutants due to their high surface area, porous structure, and strong adsorption capacity.^22–24^ In photocatalytic systems, carbon materials can serve multiple roles, including concentrating pollutants near reactive sites, acting as the center to temporarily capture excited electron to suppress electron–hole (e^−^/h^+^) recombination, and enhancing light absorption.^19^ The combination of ferrite photocatalysts with carbon supports has shown synergistic effects, leading to improved adsorption–photodegradation performance and higher overall treatment efficiency.^25^

This work demonstrated the effective degradation of a highly persistent and emerging contaminant PFOS using a cost-effective and structurally simple photocatalyst based on MnFe_2_O_4_ (FMO). Instead of employing complex structural modification or bandgap engineering strategies, this study emphasizes enhancing the practical degradation performance of a conventional ferrite material, MnFe_2_O_4_ toward an environmentally relevant and recalcitrant pollutant. To enhance catalytic efficiency, commercial AC was incorporated as a supporting matrix for the dispersion of FMO nanoparticles and promote the interfacial interaction. The obtained FMAC composite was systematically characterized and evaluated for PFOS degradation under light irradiation, with particular emphasis on catalytic performance, stability, recyclability, and the fundamental degradation mechanisms.

## 2. Experiment

### 2.1. Reagents

Fe(NO_3_)_3_·9H_2_O, Mn(NO_3_)_2_·4H_2_O, HCl, NaOH, and polyvinyl alcohol (PVA), were supplied by Shanghai Macklin Biochemical Technology Co., Ltd (China). AC was domestically purchased from Tra Bac Jsc. (Vietnam) (detail in Table S1). Methanol (MeOH), ammonium acetate (CH_3_COONH_4_), AgNO_3_, ethylenediaminetetraacetic acid (EDTA), isopropyl alcohol (IPA), and 1,4-benzoquinone (BQ) were provided by Sigma-Aldrich. PFOS (100 µg L^−1^) was supplied by AccuStandard (USA). All chemical reagents at analytic grade were used without additional purification.

### 2.2. Materials preparation

FMO and FMAC were synthesized by the gel combustion method, according to a previously reported study.^26^ For the preparation of FMAC composite, 4.5 mL of Fe(NO_3_)_3_ 0.5 mol L^−1^ solution and 0.5 mol of Mn(NO_3_)_2_ 0.5 mol L^−1^ solution were gently added into a 100 mL beaker containing 20 mL of an as-prepared PVA 5 wt% solution. The mixture was kept agitated at 80 °C using a magnetic stirrer for 15 min. Afterwards, 1.0 g of AC was dispersed into the solution and stirred vigorously for the following 2 hours to obtain wet black gel. The gel was subsequently atmospheric dried at 100 °C for 8 hours before grinding in an agate mortar and then calcined at 300 °C for 2 hours to obtain the final dark powder. FMO was prepared with the same procedure without the addition of AC support.

### 2.3. Materials characterization

Thermal behavior of FMAC sample was firstly examined in the Labsys Evo equipment by SETARAM (France). The microstructure and morphology of the prepared materials was then investigated *via* electron microscopies, including a Hitachi S4800 (Japan) scanning electron microscope (SEM) and a JEM 2100 (Japan) transmission electron microscope (TEM). The elemental composition and mapping of FMAC was obtained using energy-dispersive X-ray spectroscopy (EDX), which was integrated with SEM system. In addition, the crystal structure and phase purity of the synthesized FMO and FMAC samples were analyzed *via* X-ray diffraction (XRD) employing a Bruker D8 Advance diffractometer (Germany) with Cu Kα radiation. The mean crystallite size was calculated from the XRD patterns employing the Scherrer equation.^27^ Furthermore, the Fourier-transform infrared (FTIR) spectra were obtained at ambient temperature utilizing a Shimadzu FTIR spectrometer (Japan) within 4000–400 cm^−1^ range. For FTIR measurement, the materials were pelletized with KBr powder while the generated data were calibrated with KBr background. On the other site, the oxidation states of the elements in the catalysts were explored *via* X-ray photoelectron spectroscopy (XPS) in an ESCA-3400 apparatus (Shimadzu, Japan) with a irradiation source of Mg Kα (1253.6 eV). The binding energy (BE) was calibrated by the Au 4f_7/2_ peak at 84.0 eV whereas the peak fitting was performed utilizing XPSPEAK41 software, employing Shirley background subtraction and Gaussian–Lorentzian functions. To follow, the surface areas of materials were derived from the data of the nitrogen adsorption/desorption at −196 °C measured in an Autosorb IQ Station based on the Brunauer–Emmett–Teller (BET) equation. Additionally, the light absorption nature of the samples was examined by diffuse reflectance spectroscopy (DRS) method using a UV-vis spectrophotometer (JASCO V-500 model, Japan). Magnetic characteristics were assessed using a vibrating sample magnetometer with a sensitivity of 10^−4^ emu, under an applied magnetic field ranging from −12 to 12 kOe, across a temperature spectrum of 77–1000 K. The surface charge of materials was examined *via* the zeta potential measuring at 25 °C using a Malvern zeta potential analyzer (England). Electrochemical characteristics, including the photocurrent response (PCR) and electrochemical impedance spectroscopy (EIS), were assessed in a Biologic SP-300 potentiostat (French). A glassy carbon electrode (1 cm × 1 cm) covered with the photocatalyst, a platinum wire, and an Ag/AgCl electrode were served as the working electrode, the counter electrode, and reference electrodes, respectively, whereas Na_2_SO_4_ 0.5 mol L^−1^ was employed as the electrolyte.

### 2.4. Photocatalytic degradation of PFOS

The photodegradation of PFOS was carried out using the equipment provided by ACE Glass Inc. (U.S.A.). The system consists of a low-pressure mercury vapor lamp 450 W immersed in a Pyrex-glass reactor. The temperature was retained by a water stream covering outside of the reactors. Before each reaction, 250 mL PFOS solution with the addition of photocatalyst was kept stirred in the darkness for 4 hours to reach the equilibrium of PFOS adsorption/desorption on examined materials. The reaction conditions, including the pH (3–11), the catalyst dosage (3, 7, 10, 15, and 20 mg L^−1^), and the PFOS initial concentration (100, 300, and 500 ppb), were adjusted for evaluation. After a certain time, 1 mL of reacted samples were collected from the reactor, isolated from solid residues by centrifugation, and then analyzed on a high-performance liquid chromatography coupled with mass spectroscopy system (HPLC/MS) (Agilent Technologies) for the determination of the remaining PFOS concentration. The operation of HPLC/MS system was programmed as in Table S2.^28^ The degradation efficiency of PFOS at time *t* is calculated using the following equation (eqn (1))1
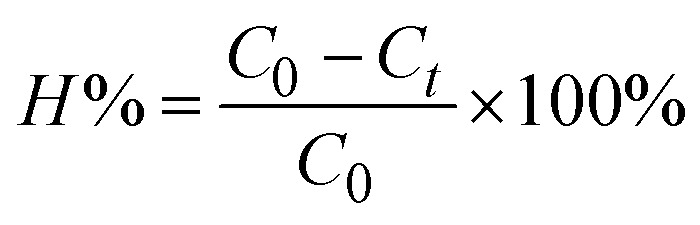
where *C*_0_ and *C*_*t*_ are the concentration of PCP at 0 and *t* min, respectively. In addition, the pseudo-first-order kinetic model was employed to determine the apparent PFOS photodegradation rate *k*_app_ (eqn (2)):2
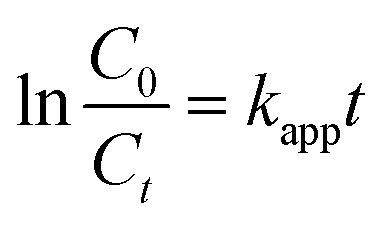


## 3. Results and discussion

### 3.1. Materials characterization

**Fig. 1:**
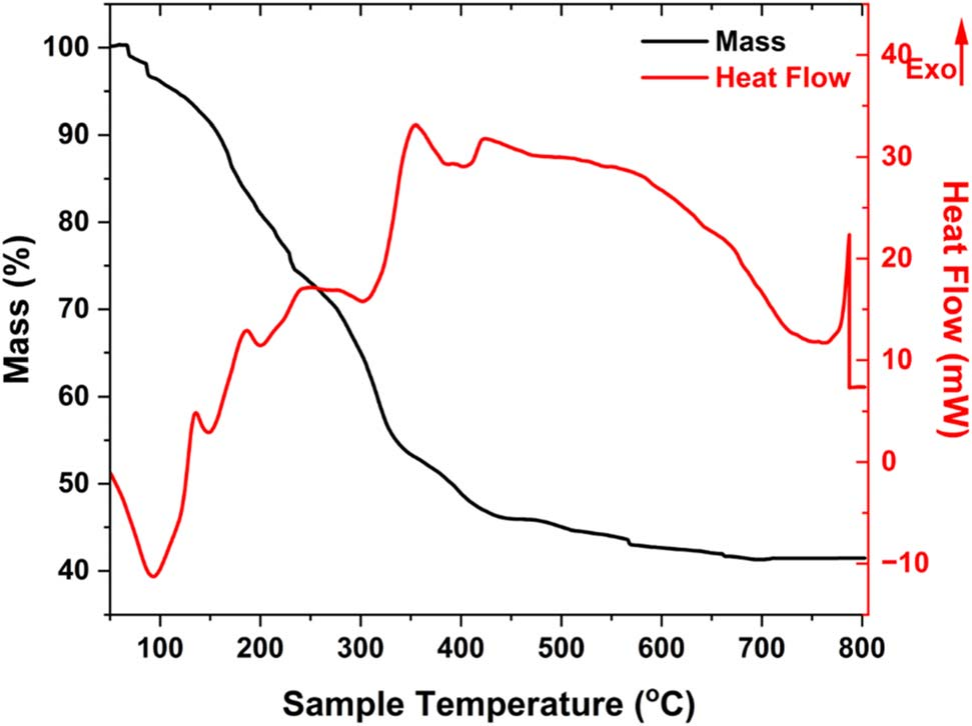
Thermal analyses of FMAC sample.

Thermal behaviors of obtained FMAC were first considered (Fig. 1). TGA curve displayed three distinct stages of mass loss. At the temperature below 150 °C, a slight loss in conjunction with a weak endothermic signal was aligned to the evaporation of physically absorbed water on the surface of materials. As the temperature increased from 150 °C to 350 °C, the sample experienced a significant mass loss due to the combustion of residues of precursors, the decomposition of oxygen-containing groups on AC, and the degradation of amorphous carbon fractions. The exothermic peaks in this interval demonstrated on DSC curve confirmed those combustions. The mass loss continued until 600 °C with a slower rate and the appearance of broad exothermic signal, which was assigned to the oxidation of graphitic-like carbon domain in AC, the decomposition of strong bound carbon. FMAC became stable after 600 °C as the change in mass was insignificant. The remaining mass was attributed to stable FMO. An exothermic signal near in DSC curves at 800 °C might be due to additional recrystallization of FMO nanoparticles.^29,30^

**Fig. 2:**
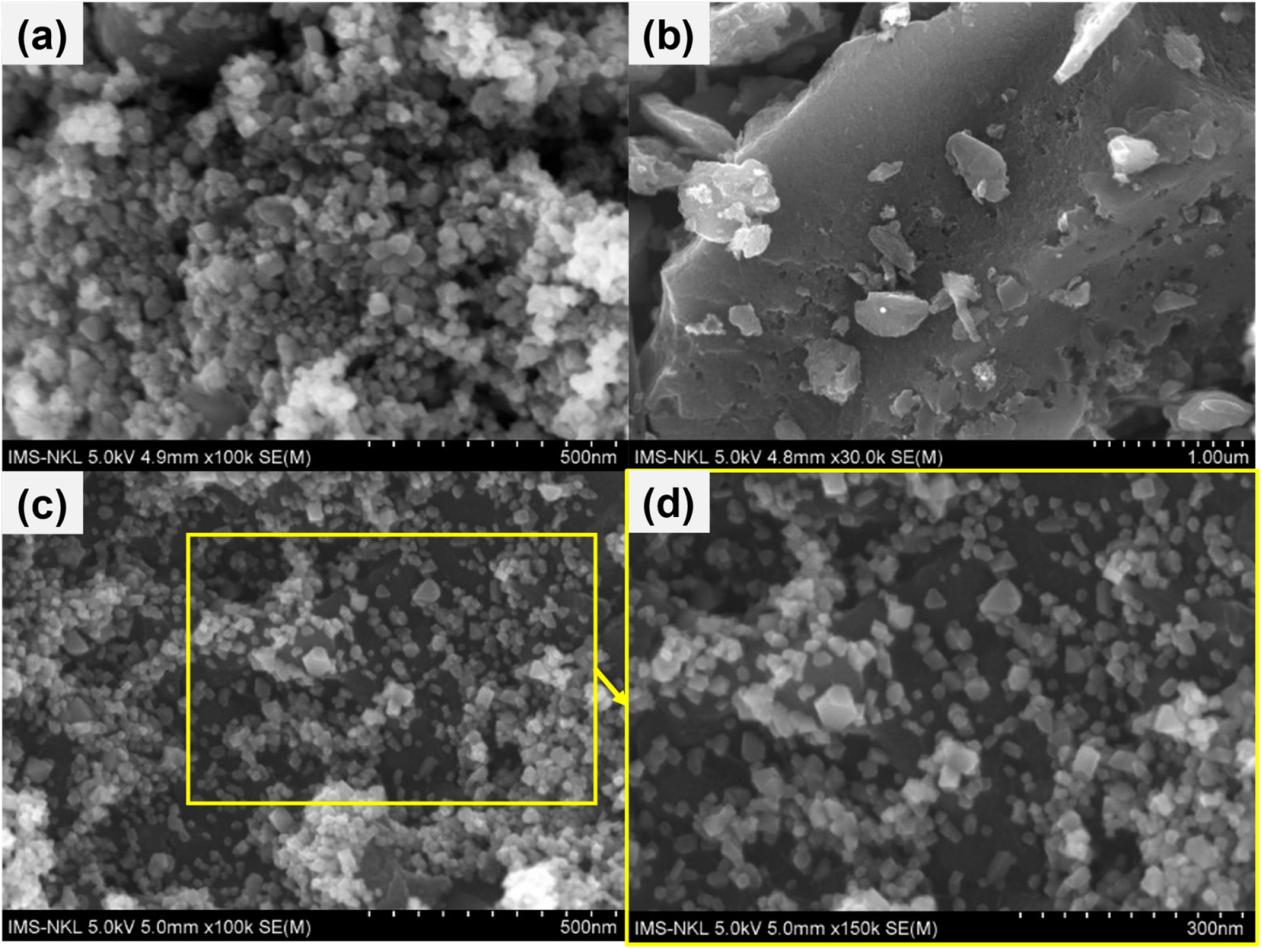
SEM images of (a) FMO, (b) AC, and (c and d) FMAC samples.

**Fig. 3:**
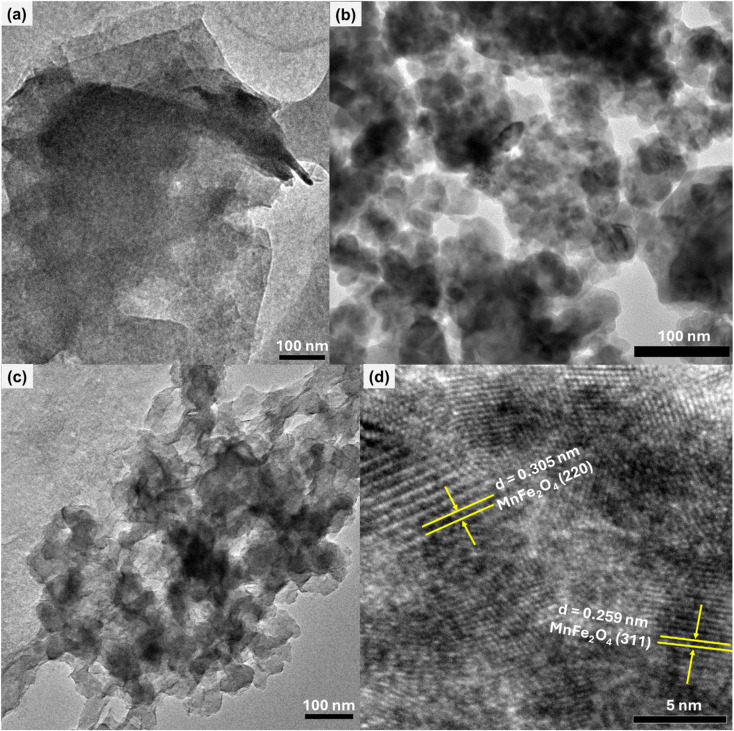
TEM images of (a) AC, (b) FMO, and (c) FMAC samples. (d) HR-TEM image of FMAC sample.

The morphology of the synthesized FMO, FMAC, and AC support was systematically investigated using SEM (Fig. 2) and TEM (Fig. 3). As shown in Fig. 2a, the pristine FMO sample consisted of uniformly shaped nanoparticles with a size of <50 nm. These nanoparticles exhibited a characteristic cubic-like morphology, which is typical of the spinel ferrite crystal structure. However, noticeable particle agglomeration was observed, resulting in the formation of dense and porous clusters. This agglomeration can be attributed to the high surface energy and magnetic interactions inherent to ferrite nanoparticles. After immobilization onto the activated carbon support, a distinct morphological evolution was observed. As shown in Fig. 2c, the FMO nanoparticles were well distributed on the surface of AC with significantly reduced agglomeration. This improvement was mainly ascribed to the high specific surface area of AC and the strong interfacial interactions between FMO nanoparticles and the surface functional groups of the carbon matrix during crystal growth. Importantly, the dispersion on AC does not alter the original cubic morphology of FMO (Fig. 2d), indicating that the spinel crystal structure was well preserved in the composite.

The TEM results further supported the SEM observations. As shown in Fig. 3a, the AC support exhibited a thin, sheet-like morphology with an amorphous structure, confirming its role as a suitable carrier. In contrast, Fig. 3b reveals that FMO nanoparticles are severely agglomerated, while retaining their cubic shape. Notably, the FMAC composite (Fig. 3c) shows homogeneous dispersion of FMO nanoparticles across the surface of AC, demonstrating that the carbon support effectively suppressed nanoparticle agglomeration. This enhanced dispersion increased the exposure of accessible catalytic/adsorptive sites, which is beneficial for catalytic/adsorption-related applications. The HR-TEM image of FMAC (Fig. 3d) displayed well-resolved lattice fringes with interplanar spacings of approximately 0.305 nm and 0.259 nm, corresponding to the (220) and (311) crystallographic planes of spinel FMO, respectively. The presence of clear lattice fringes confirmed the high crystallinity of the FMO nanoparticles within the composite. Meanwhile, the surrounding regions are attributed to the amorphous AC matrix, further verifying the successful decoration of amorphous AC support with crystalline FMO nanoparticles.

**Fig. 4:**
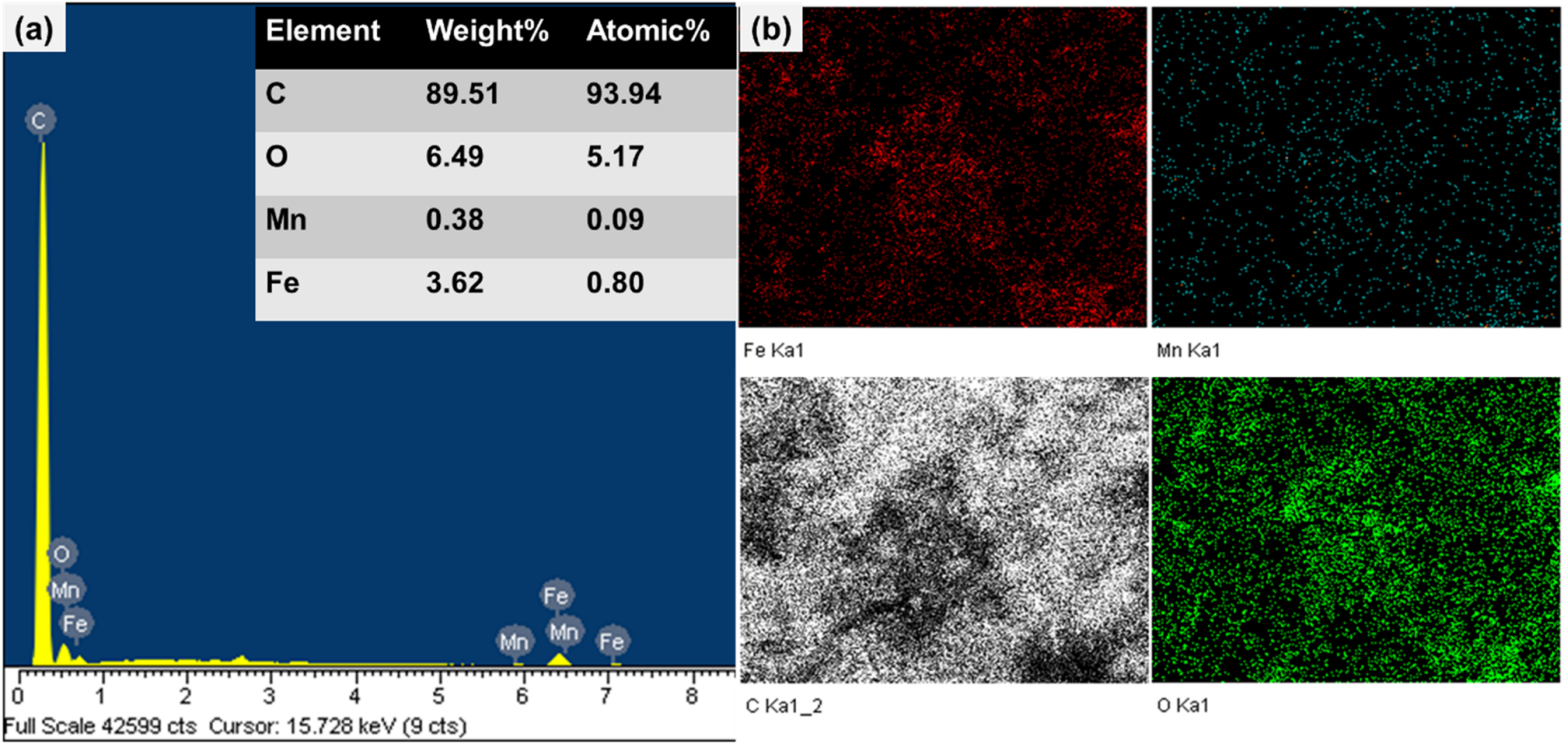
(a) EDX spectrum and (b) elemental mapping of FMAC sample.

The elemental composition and distribution of FMAC sample was then analyzed and discussed. The EDX spectrum (Fig. 4a) confirmed the presence of C, O, Mn, and Fe, verifying the successful loading of FMO onto the AC support. Carbon was the dominant component (89.51 wt%), originating from AC, while oxygen (6.49 wt%) was as a part of the ferrite lattice and surface functional groups. The Mn and Fe contents of 0.38 and 3.62 wt%, respectively, exhibited an atomic ratio consistent with the initial Mn/Fe precursor stoichiometry, indicating good compositional preservation. Elemental mapping of FMAC in Fig. 4b revealed a uniform distribution of Mn and Fe across the carbon matrix, confirming homogeneous dispersion of FMO nanoparticles on AC carrier throughout the composite.

**Fig. 5:**
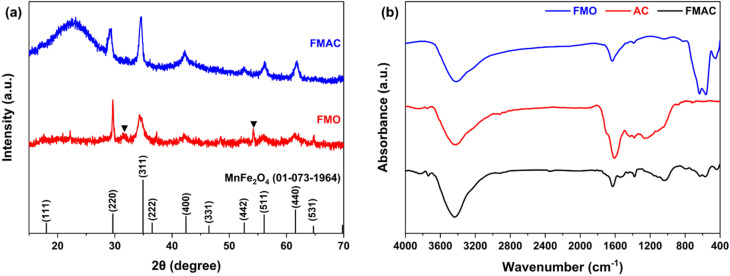
(a) XRD and (b) FTIR analyses of prepared FMO and FMAC samples.

Fig. 5a demonstrated the XRD patterns of as-prepared FMO and FMAC samples. FMO curve exhibited the diffraction peaks associated with the characteristic crystal planes of spinel MnFe_2_O_4_, including the (111), (220), (311), (222), (400), (331), (422), (511), (440), and (531) reflections, which was in agreement with the standard JCPDS card no. 01-073-1964.^31,32^ However, additional peaks of impurity were still presence at the 2*θ* of 31.55° and 54.23°. Those ▼-marked diffractions were attributed to the presence of Fe_2_O_3_ phase in FMO samples.^33^ For the FMAC composite, the diffraction peaks corresponding to FMO are still clearly visible, indicating that the crystalline structure of FMO is well preserved after loading onto the activated carbon support. In addition, a broad and weak diffraction feature in the low-angle region (centered at 22.75°) was observed, which can be ascribed to the amorphous phase of AC.^34^ The relatively reduced intensity of FMO peaks in FMAC compared to reference FMO was mainly due to the dilution effect of the carbon matrix and the good dispersion of ferrite nanoparticles on the AC surface. In addition, the corresponding Fe_2_O_3_-phase was not observed in FMAC curves, possibly due to the presence of AC as reductive agent to prevent the oxidation to Fe_2_O_3_.^35^ Moreover, no other strange signal was detected on FMAC diffraction curve. By using the Scherrer equation,^36^ the size of FMO and FMAC derived from XRD data was calculated, resulting in the values of 6.37–37.27 nm and 3.43–12.82 nm, respectively. This calculation once revealed the role of AC on reducing and controlling the size of MFO nanoparticles in the composite.

Fig. 5b exhibited the FTIR spectra of AC, FMO, and FMAC sample, providing information on surface functional groups and interfacial interactions. The FTIR spectrum of AC exhibits a broad absorption band near 3400 cm^−1^, which related to the stretching vibration of surface –OH groups or adsorbed moisture. The bands observed in the range of 1600–1700 cm^−1^ was assigned to C=O stretching vibrations of oxygen-containing functional groups, while the peaks between 1000 and 1200 cm^−1^ were related to C–O stretching vibrations, confirming the presence of abundant surface functionalities on activated carbon. The FMO spectrum shows characteristic absorption bands in the low-wavenumber region, typically below 600 cm^−1^, which are attributed to the stretching vibrations of metal–oxygen bonds (Mn–O and Fe–O) in the tetrahedral and octahedral sites of the spinel ferrite structure,^37,38^ confirming the formation of FMO nanoparticles. Additional low-absorbance peaks between 800 and 1700 cm^−1^ on FMO curve were stilled observed, which were ascribed to organic bonds or functional groups of PVA residues.^39^ In the curve of FMAC composite, both the characteristic bands of AC and FMO were presented, indicating the coexistence of activated carbon and FMO phases. Notably, slight shifts and intensity changes in the –OH and C–O related bands were observed, suggesting interfacial interactions between the ferrite nanoparticles and AC support *via* attached oxygen-containing groups.

**Fig. 6:**
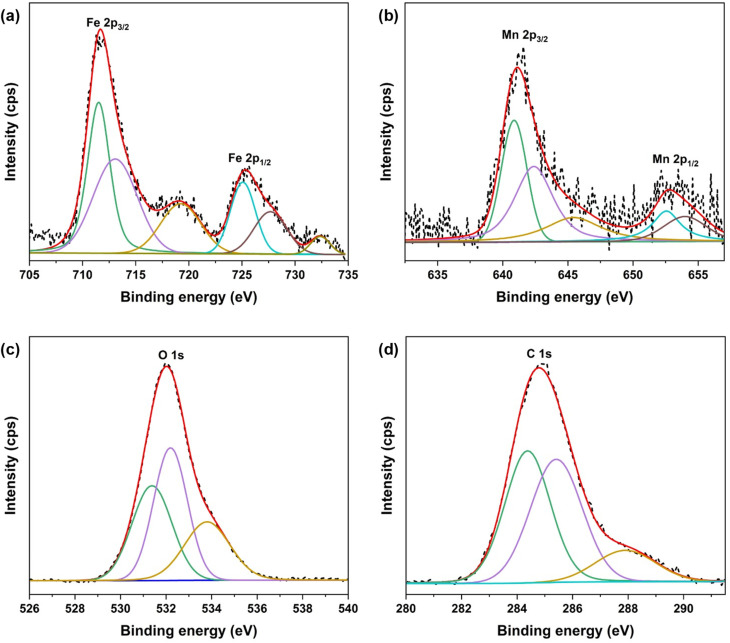
XPS spectra of (a) Fe 2p, (b) Mn 2p, (c) O 1s, and (d) C 1s orbitals in MFAC sample.

XPS technique was then employed to examine the oxidation state of include elements in MFAC composite, resulting in the presence of Fe, Mn, O, and C, aligning with EDX results. Fig. 6 displayed the detailed core level spectra of Fe 2p, Mn 2p, O 1s, and C 1s orbitals. The Fe 2p spectrum was presented Fig. 6a where two main peaks centered at around 711.7 eV and 725.4 eV were assigned to Fe 2p_3/2_ and Fe 2p_1/2_, respectively. The Fe 2p_3/2_ and Fe 2p_1/2_ were deconvoluted into four different peaks, suggesting the existence of both Fe^2+^ and Fe^3+^ in FMAC composite and in agreement with previous results of MnFe_2_O_4_ spinel.^40^ The shake-up satellite peaks located at 719.2 eV and 732.4 eV were observed, which was ascribed to the presence of Fe^3+^ in the α-Fe_2_O_3_.^41^ The doublet peaks located at 641.2 eV and 652.8 eV for Mn 2p orbital were observed in Fig. 6b. Those signals corresponded to Mn 2p_3/2_ and Mn 2p_1/2_ levels, respectively, were split into five ones at 640.8 eV (*m*_1_), 642.4 eV (*m*_2_), 645.4 eV (*m*_3_), 652.5 eV (*m*_4_), and 654.0 eV (*m*_5_). While *m*_1_ and *m*_4_ confirmed the presence of Mn^2+^, *m*_2_ and *m*_5_ stated the occurrence of Mn^3+^.^42^ Moreover, the satellite peak *m*_3_ arised the formation of MnO_2_ in FMAC sample.^42,43^ The multiple oxidation state nature of both Mn and Fe in the composite would be advantages for the occurrence of additional catalytic oxidation for PFOS treatment in conjunction with the principal photocatalytic processes.^44,45^

The XPS spectrum of O 1s orbital resulted in a peak centered at 532.0 eV, which was the overlap of three different peaks (Fig. 6c). While the deconvoluted signal at 531.4 eV described the oxygen–metal (Mn–O and Fe–O) bonds, other higher-BE signals at 532.2 eV and 533.8 eV represented for the oxygen vacancies or absorbed water/hydroxide groups, and the carbonate species (C–O or C=O groups), respectively.^46^ Similarly, the spectrum of C 1s orbital might also be deconvoluted into three separated peaks at 284.4 eV, 285.4 eV, and 287.9 eV, which were corresponding to the C–C/C=C bonds, C=O, and O–C=O groups^47,48^ (Fig. 6d). The presence of high abundant oxygen-containing groups as described in both O 1s and C 1s spectra provided binding sites for the immobilization of FMO nanoparticles on AC support during preparation step and further suppress particle aggregation as also described in the above SEM and TEM images.^49^

**Fig. 7:**
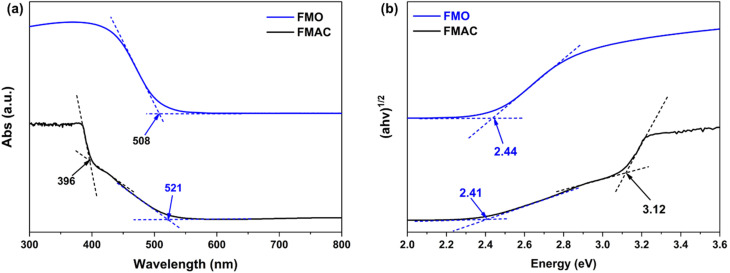
(a) DRS and corresponding (b) Tauc plot of FMO and FMAC samples.

To follow the materials characterization, DRS measurement was conducted to survey the light absorption of prepared powder (Fig. 7a). The FMO sample exhibited a strong absorption in the visible region, with a sharp absorption edge at 508 nm, which is typical for spinel ferrites and is related to O^2−^ → Fe^3+^/Mn^3+^ charge-transfer transitions and d–d electronic transitions within the ferrite lattice.^50,51^ On the other hand, as dispersed on AC support, along with the broadening absorption band of FMO with the edge at 521 nm, an additional absorption near UV light region was also observed, which was attributed to the light absorption of AC component. The direct bandgaps (*E*_g_) of the samples were then derived from DRS data using Kubelka–Munk equation (eqn (3)) and presented by a Tauc plot^52^ (Fig. 7b).

3 *αhν* = *A*(*hν* − *E*_g_)^1/2^

where *α* is the absorption coefficient, *hν* is the incident photon energy, and *A* is constant. The calculated *E*_g_ of MFO and those immobilized ones on AC had the values of 2.44 eV and 2.41 eV, respectively. The slight redshift of the apparent absorption edge as well as the alteration of *E*_g_ of FMO nanoparticles indicated the interaction between immobilized FMO nanoparticles with AC matrix.^53^ In addition, the DRS studies also demonstrated the nature of well-visible-light response of as-prepared materials that would be beneficial to photocatalytic applications.

**Fig. 8:**
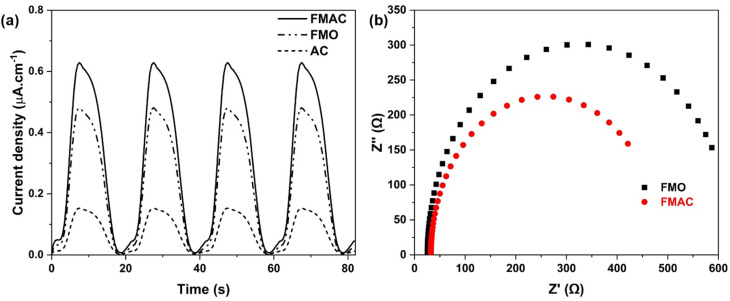
(a) PCR and (b) Nyquist plots for EIS using FMO and FMAC samples.

In the next step, the charge transfer on the surface of FMO-based materials were then examined using electrochemical techniques, including PCR and EIS. In Fig. 8a, the photocurrent–time curve of FMO showed a much higher density than AC and sharply enhanced as FMO dispersed on AC support. To supplement, the EIS Nyquist plot demonstrated a lower charge-transfer resistance (*R*_ct_) of FMAC composite as compared to FMO (Fig. 8b). The results indicated that not only were more charged species generated but also the mobility or charge transfer on the surface of materials were significantly facilitated. The data also suggested the recombination of charge carriers was remarkably limited on FMAC composite, which would be further beneficial to photocatalysis. The enhancement was contributed by the presence of oxygen-containing functional groups as well as the presence of unsaturated π bonds as excited electron traps in AC. However, the PCR curves also described an unstable current of all prepared materials as the photocurrent signficantly drop after reaching the peaks.^54,55^

**Fig. 9:**
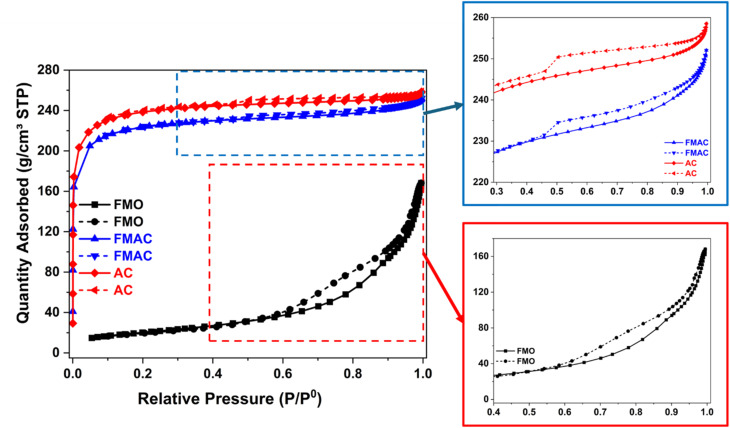
Nitrogen adsorption/desorption isotherms of FMO, FMAC, and AC samples.

A larger surface area material generally leads to higher photocatalytic activity because of a high density of active sites for catalysis.^56^ The photocatalysts with high specific surface area can adsorb more reactant molecules, improve light harvesting by providing more reactive interface, and facilitate charge transfer at the surface, thereby enhancing overall photocatalytic efficiency. The nitrogen adsorption/desorption isotherms (N_ad/des_) in combination with BET equation were employed to determine the surface area of prepared samples. Fig. 9 showed the behaviors of as-prepared materials for N_ad/des_ investigation. All materials illustrated the type IV isotherms with H4 hysteresis loops, possessing the presence of both micropores and mesopores with slit-like pore geometry of examined samples.^57^ While the hysteresis loop of FMO started at a relative pressure (*P*/*P*^0^) of near 0.55, those of AC and FMAC started to appear at *P*/*P*^0^ of 0.4. Subsequently, the calculated *S*_BET_ of those materials was increased in the order of FMO (77.64 m^2^ g^−1^) < FMAC (698.65 m^2^ g^−1^) < AC (733.67 m^2^ g^−1^). The obtained results indicated a significant enhancement by nine times in *S*_BET_ of FMAC composite as compared to FMO, suggesting the slight reduction in *S*_BET_ of AC as the oxide dispersed on was due to the filling in the pores and the surface occupation of carriers by FMO nanoparticles as well as the partly collapse of porous structure during FMAC preparation.^58^ Overall, the adsorption behavior once confirmed the successful integration of MnFe_2_O_4_ onto the high-surface-area AC templates, preserving a hierarchical micro/mesoporous structure that can enhance surface accessibility and mass transport for adsorption-enhanced photocatalysis.

**Fig. 10:**
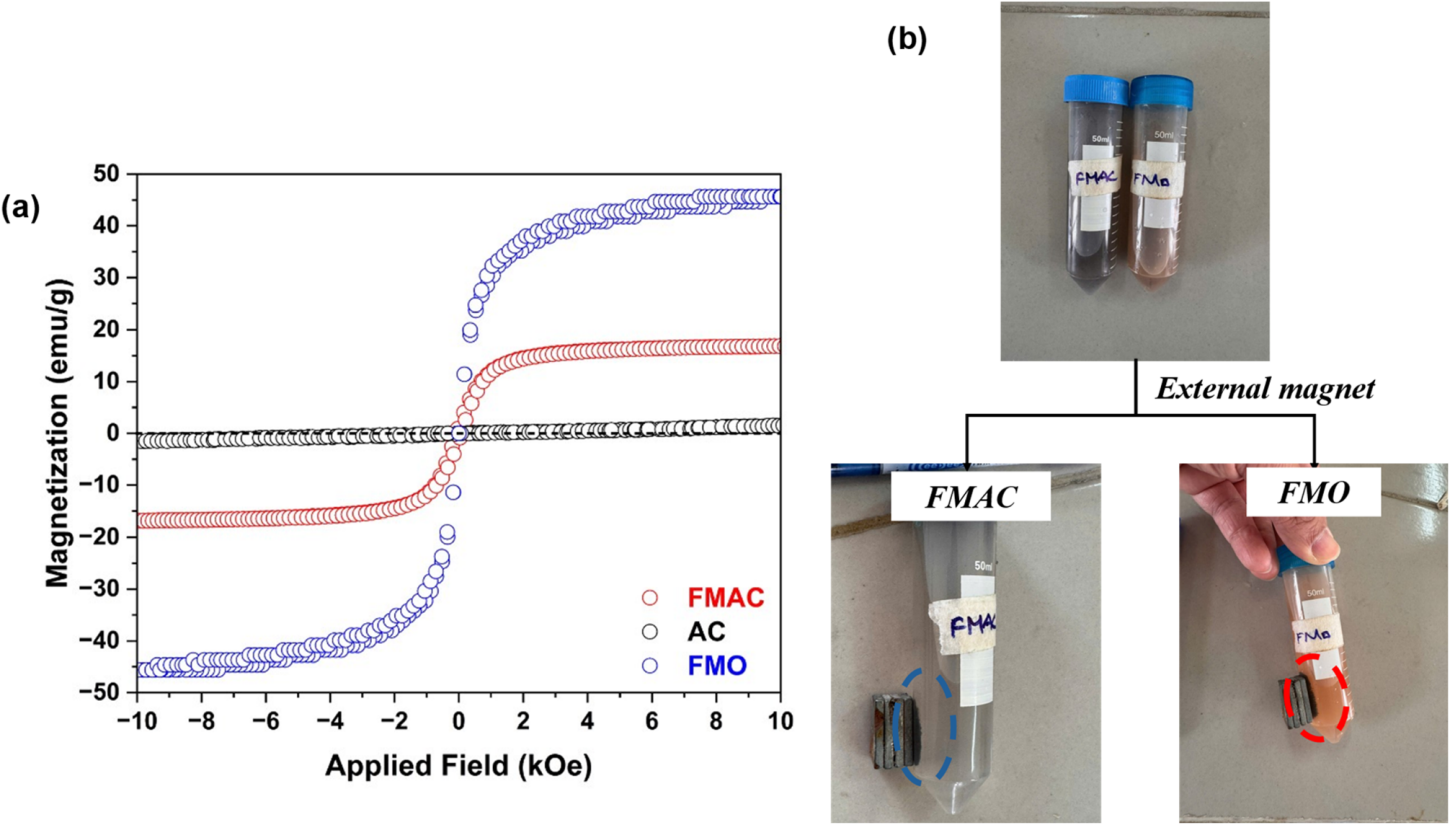
(a) VSM and (b) recovery tests from water of FMAC and FMO.

The magnetic properties of the prepared samples were evaluated by VSM (Fig. 10a). The hysteresis loops indicate that FMO and FMAC exhibit typical near-supermagnetic nature at room temperature, commonly observed for spinel ferrite nanoparticles with small particle size.^59^ Compared with FMO (45.60 emu g^−1^), the saturation magnetization of FMAC (16.90 emu g^−1^) was significantly reduced after loading onto AC, which was hardly magnetized. This decrease can be attributed to the dilution effect of the non-magnetic carbon matrix or surface spin disorder at the ferrite–carbon interface. Nevertheless, FMAC still retained sufficient magnetic responsiveness, which was critical for its practical application to recover that composite (Fig. 10b) to effectively limit the release of secondary pollutants associated with nanoparticle leaching into the aquatic environment. Therefore, the combination of photocatalytic functionality and magnetic separability makes the FMAC composite a promising and environmentally friendly material for wastewater remediation.

### 3.2. Investigation on photocatalytic degradation of PFOS

**Fig. 11:**
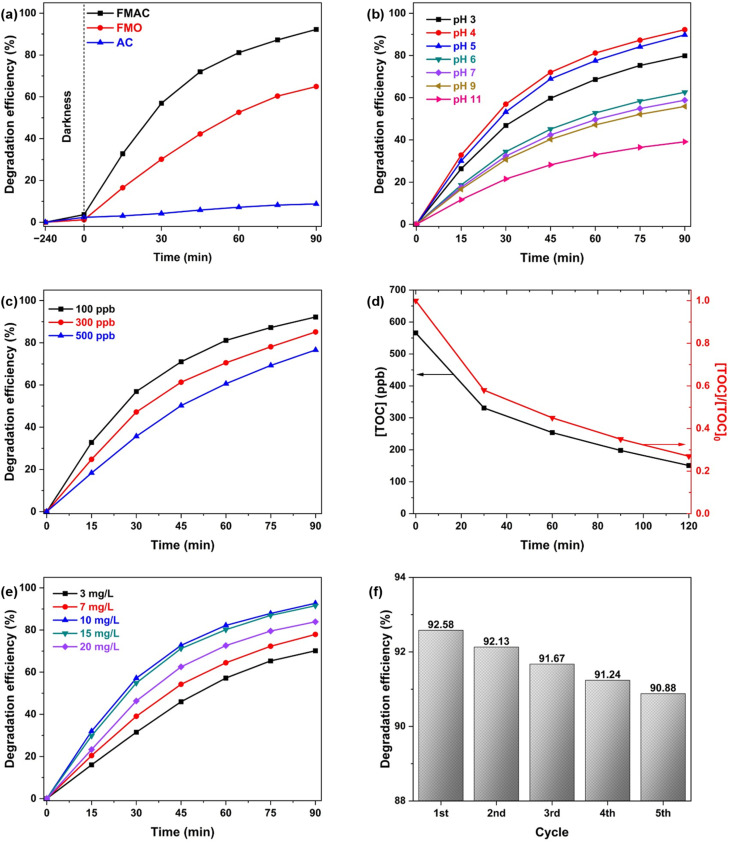
Effects of factors on PFOS photodegradation: (a) photocatalysts, (b) pH, (c) PFOS initial concentrations, (e) FMAC dosage. (d) TOC changes (with PFOS 300 ppb) and (f) durability tests of PFOS (100 ppb) photodegradation using FMAC.

The photocatalytic degradation of PFOS using FMAC was initially compared to those of FMO and AC for the first set of experiments (Fig. 11a). In this set, PFOS 100 ppb solutions were prepared and adjusted to be acidic at pH 4.0 in presence of 10 mg L^−1^ of catalysts. Before illumination, the adsorptive treatment of PFOS using AC, FMO, and FMAC in darkness for 4 hours showed the negligible removal efficiencies of 2.36%, 1.19%, and 3.65%, respectively. When turning on the light source, while AC scarcely demonstrated their photocatalytic activity for PFOS degradation, FMO could lower the initial PFOS concentration by a half after the first hour. On the hand, FMAC composite exhibited its extraordinary effectiveness for PFOS degradation with a DE value of 81.15% in the first 60 min. As the degradation time was prolonged to 90 min, FMAC, FMO, and AC could reach a DE value for PFOS degradation of 92.21%, 64.93%, and 8.78%, respectively. Subsequently, the pseudo-first-order kinetic model was utilized to elucidate the apparent rate *k*_app_ of PFOS degradation using those photocatalysts (Fig. S1a). The calculated *k*_app_ values (with the linear regression *R*^2^ ≈ 0.99) were increased in the order of AC (0.0012 min^−1^) < FMO (0.0120 min^−1^) < FMAC (0.0280 min^−1^). The investigation for photocatalytic treatment of PFOS using different prepared materials was as expected and in agreement with above materials characterization. FMO possibly remediates PFOS at a moderate level since this catalyst faces the challenge of the particle aggregation that hides or reduces the active site, and the high electron/hole recombination rate. Meanwhile, AC hardly photodegrades PFOS under examined conditions and the reduction in PFOS concentration is mainly from adsorptive removal. On the other, FMAC composite with a strong synergistic effect between FMO and AC overcomes the limitation of barely FMO for PFOS degradation by surpassing the particles aggregation at nanoscale to release the more catalytic centers as well as promoting the charge separation and the additional adsorption of pollutants onto the photocatalyst.

In the following series of experiments, the factors that commonly affect the PFOS photodegradation, including the pH, photocatalyst dosage, and the initial PFOS concentration were carefully evaluated. Fig. 11b illustrated the influence of initial solution pH on the photocatalytic degradation performance of PFOS at a concentration of 100 ppb using FMAC 10 mg L^−1^. The PFOS DE using FMAC was strongly pH-dependent since it reached the highest DE of under acidic conditions of pH 4.0 (92.21%) to 5.0 (89.77%) before gradually decreased as the PFOS solution became more alkaline of pH 11.0 (39.10%). Since the reported p*K*_a_ of PFOS is around −3.27,^60^ the major form of PFOS in this work is anionic. Under acidic conditions, as the FMAC composite surface was positively charged (Fig. S1b), the adsorption of PFOS onto the catalysts' surface was remarkably enhanced by electrostatic force, facilitating the contact between PFOS and FMO catalytic centers for pollutant degradation. Additionally, acidic environments favor the formation of reactive oxygen species such as ˙OH radicals, further promoting photocatalytic reactions. A higher pH value, above the isoelectric point (IEP) of 5.8, caused the occurrence of electrostatic repulsion between the catalyst and PFOS anions. As a result, the limited contact between the catalytic centers (or the generated oxidative species) led to the less effective PFOS photodegradation. Moreover, alkaline media of pH 9.0–11.0 could even scavenge the active radicals.

The effect of the initial PFOS concentration was then studied (Fig. 11c). While the pH of PFOS solution was adjusted to 4.0 in the presence of FMAC 10 mg L^−1^, the pollutant concentrations were controlled at 100 ppm, 300 ppm, and 500 ppm. FMAC showed the most effectiveness for FPOS treatment at a concentration of 100 ppb (92.21%) and gradually decreased with increasing the initial PFOS concentration, although FMAC still maintained the relatively high removal efficiency of 85.15% (300 ppb) and 76.59% (500 ppb). Since the PFOS did not absorb light energy under established conditions,^61^ the reduction in DE was due to the overload of active sites for PFOS remediation. As concentration increased, more pollutant molecules competed for the same number of active sites, leading to partial saturation of the surface. Moreover, additional generated intermediates accumulated surrounding the active sites also inhibited the contact between PFOS and FMO for the activation of the degradation processes. The occurences of intermediates were confirmed by mineralization studies, which were evaluated by the variation of TOC values for the experiment with PFOS concentration of 300 ppb (Fig. 11d). The [TOC]/[TOC]_0_ could drop to 0.35, corresponding to a mineralization of 65%, after 90 min of reaction, and possibly reached 73% after 120 min, which was lower than the corresponding DE value. It is suggested that FMAC is effective in breaking down complex organic molecules, but complete mineralization requires additional irradiation times.

Fig. 11e illustrates the influences of FMAC dosage on PFOS DE. The increase in the catalyst dosage from 3.0 to 10.0 mg L^−1^ significantly enhances the treatment effectiveness from 70.14% to 92.69% (in 90 min), which can be attributed to the increased number of available active sites and improved light absorption. However, further increasing the catalyst dosage (15 mg L^−1^) resulted in only slight improvement to 93.25%, and even decreased to 83.92% as the composite content reached 20 mg L^−1^. This behavior may be due to light scattering and shielding effects at higher catalyst loadings, which reduced the effective penetration of light into the suspension or the catalytic sites, on the other hand, were hidden by a high amount of AC support. Therefore, a MFAC content of 10 mg L^−1^ ensured the PFOS remediation under the conditions of this study.

Finally, the reusability of FMAC photocatalyst for PFOS treatment in water was tested for several cycles. pH, the PFOS concentration, and FMAC dosage were adjusted to 4.0, 100 ppb, and 10 mg L^−1^, respectively, for this examination. After each cycle, PFOS concentration and pH were measured and re-adjusted to the initial conditions to simulate a pseudo continuous treatment process. The degradation efficiency shows only a trivial decrease from 92.58% in the first cycle to 90.88% in the fifth cycle without altering the catalyst's structure (Fig. S2), indicating good stability and durability of FMAC for PFOS photodegradation. The minor loss in photocatalytic activities might be related to partial surface fouling by reaction intermediates or slight catalyst loss by the environment during treatment process. Combined with its magnetic recoverability (Fig. 10), FMAC demonstrates strong potential for practical PFOS treatment applications with minimal risk of secondary pollution.

### 3.3. Study on mechanism of PFOS photodegradation using FMAC

Firstly, the CB and VB energy potential can be derived from the following equations^62^ (eqn (4) and (5))

4 *E*_VB_ = *χ* − *E*^e^ + 0.5 × *E*_g_

5 *E*_CB_ = *E*_VB_ − *E*_g_

where *χ* is the electronegativity of the semiconductor in Mulliken scale. For MnFe_2_O_4_, *χ* is 5.99 eV. *E*^e^ is the free electron energy on the hydrogen electrode scale (4.5 eV), while *E*_CB_ and *E*_VB_ are the energies of CBs and VBs, respectively. From DRS studies, with *E*_g_ in the composite of 2.41 eV, the calculated *E*_CB_ and *E*_VB_ were 0.29 eV and 2.70 eV, respectively.

**Fig. 12:**
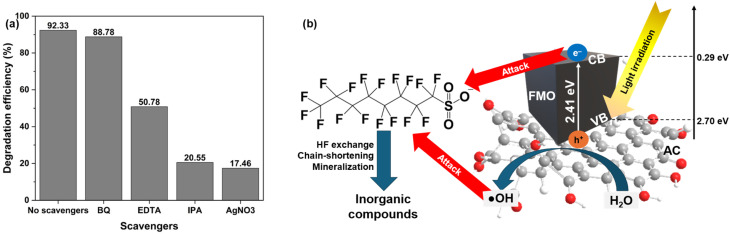
(a) Scavenger test and (b) proposed degradation mechanism using FMAC photocatalyst.

Secondly, the radical-trapping experiments that provided direct insight into the dominant reactive species and the charge-transfer pathway responsible for pollutant degradation over the FMAC photocatalyst were conducted (Fig. 12). As shown in Fig. 12a, PFOS degradation remained a high DE in the absence of scavengers and slightly reduced from 92.33% to 88.78% by the addition of BQ as superoxide radicals. This observation indicated that ˙O_2_^−^ plays a negligible role in the degradation process. This was consistent with the electronic structure of MnFe_2_O_4_, since *E*_CB_ was relatively more positive than the standard redox potential of ˙O_2_^−^/O_2_ and the reaction O_2_ + e^−^ → ˙O_2_^−^ was thermodynamically unfavorable. As a result, photogenerated electrons are unlikely to participate in superoxide radical formation, explaining the weak influence of BQ. In contrast, the introduction of hole scavengers, EDTA reagent, halved the DE of FPOS photodegradation (50.78%). Since *E*_VB_ of FMO was more positive than the standard redox potential of H_2_O/˙OH, h^+^ possibly interact with H_2_O for the generation of hydroxyl radicals ˙OH, which was the major reactive species in photocatalytic degradation of diverse organic substances. In addition, Duan *et al.* suggested that h^+^ could also react directly with PFOS for the formation of other radicals, like ˙C_8_H_17_ and ˙SO_3_^−^ for the subsequent chain reactions.^63^ Furthermore, both ˙OH (IPA) and e^−^ (AgNO_3_) scavengers nearly halted the PFOS degradation as the DE values reached the bottoms of 20.55% and 17.46%, respectively. The results were in agreement with previous studies since ˙OH and e^−^ were well-described as the major species taking part in the defluorination (H/F exchange), desulfonation, (de)carboxylation, chain-shortening, and ultimately mineralizing the PFOS molecules.^64–66^

Finally, a plausible degradation mechanism was proposed (eqn (6)–(9)) and summarized in Fig. 12b. Under light irradiation, FMO catalytic centers in FMAC composite absorbed photons and generated e^−^/h^+^ pairs. The photogenerated h^+^ in the VB was the key oxidative species, either directly oxidizing the pollutant or reacting with surface-bound H_2_O to produce ˙OH radicals to degrade the organic molecules. Although, e^−^ in the CB cannot react with dissolved oxygen in the solution for the formation of ˙O_2_^−^, they still expressed their key role in analogous with ˙OH radicals in other reactions, including desulfonation, carboxylation, chain-shortening, *etc.*, toward the mineralization of PFOS. The AC matrix with multiple π bonding or oxygen-containing functional groups acted as an electron acceptor and conductive support, facilitating charge separation and suppressing the recombination, leading an enhancement of photocatalytic activity toward PFOS degradation.6
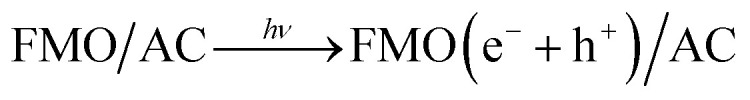

7 FMO(e^−^ + h^+^) + AC → FMO(h^+^) + AC(e^−^)

8 FMO(h^+^) + H_2_O → H^+^ + HO˙ + MFO

9 FMO(h^+^) + AC(e^−^) + HO˙ + PFOS → intermediates → inorganic products

## 4. Conclusion

This work successfully prepared FMAC composite by the gel combustion process in order to effectively overcome the intrinsic limitations of bare ferrite photocatalysts. Structural and surface analyses confirmed the homogeneous dispersion of spinel FMO nanoparticles on the carbon framework, supported by the preservation of Mn/Fe stoichiometry and effective metal–oxygen–carbon interactions. The nature of multiple oxidation states of Mn and Fe, together with abundant surface functional groups, provided favorable conditions for interfacial charge transfer and suppressed nanoparticle aggregation. Optical and electrochemical results consistently showed that the FMAC composite exhibits enhanced visible-light absorption, reduced charge transfer resistance, and improved separation of photogenerated charge carriers compared with FMO alone. The large surface area and hierarchical pore structure of activated carbon further promoted pollutant adsorption, increasing the effective contact between reactive species and target molecules. As a result, the fabrication promoted PFOS photocatalytic on FMAC composite to reach a degradation efficiency of ∼94% and a rate constant of 0.0280 min^−1^, which about twice higher than bare FMO nanoparticles. In addition, the monitoring of reaction condition indicated that the most effective treatment of PFOS was obtained at pH 4.0, an initial contaminant concentration of 100 ppb, and a FMAC content of 10.0 mg L^−1^. Later, the test for PFOS photodegradation in the presence of active radical scavengers clarified that photogenerated h^+^ and ˙OH were the main active species responsible for degradation, while ˙O_2_^−^ expressed an insignificantly role due to the relatively positive CB position of FMO. Furthermore, the magnetic nature of the composite allowed rapid separation from water, minimizing secondary pollution and improving reusability. Overall, the synergistic integration of adsorption, light harvesting, and charge-transfer functions in FMAC composite, suppressing the limitation of bare FMO nanoparticles, makes it a promising photocatalyst for water treatment applications.

## Author contributions

Nguyen Trung Kien: project administration, funding acquisition, investigation, writing – original draft; Le Bao Hung: investigation, formal analysis, resource; Nguyen Quang Bac: software, visualization; Nguyen Thi Ha Chi: investigation, formal analysis; Pham Ngoc Chuc: investigation, formal analysis; Do Nguyen Huy Tuan: investigation, formal analysis; Nguyen Tran Dung: software, visualization; Truong Minh Tri: investigation, formal analysis; Nguyen Vu Ngoc Mai: conceptualization, methodology, investigation, data curation. Dao Ngoc Nhiem: data curation, supervision, validation, writing – review and editing.

## Conflicts of interest

The authors declare no conflict of interest.

## Supplementary Material

RA-016-D6RA00405A-s001

## Data Availability

All data and materials generated or analyzed during this study are included in this published article and supplementary information (SI) and are available from the corresponding author upon reasonable request. Supplementary information is available. See DOI: https://doi.org/10.1039/d6ra00405a.
