# Supplementary material for: Decoration of MnFe2O4 nanoparticles on activated carbon as recoverable photocatalyst for perfluorooctanesulfonic acid degradation in water
Source: RSC Adv. 2026 Mar 25;16(18):16329–43. doi: 10.1039/d6ra00405a (PMC13015944; doi:10.1039/d6ra00405a)
Supplement: RA-016-D6RA00405A-s001 [file RA-016-D6RA00405A-s001.pdf]

Supplementary Information (SI) for RSC Advances.
This journal is © The Royal Society of Chemistry 2026

Supplementary document for

Decoration of $MnFe_2O_4$ nanoparticles on activated carbon as recoverable photocatayst for perfluorooctanesulfonic acid degradation in water

Nguyen Trung Kien[1,2], Le Bao Hung[2,3], Nguyen Quang Bac[1], Nguyen Thi Ha Chi[1], Pham Ngoc Chuc[1], Do Nguyen Huy Tuan[1], Nguyen Tran Dung[1,2], Truong Minh Tri[4], Nguyen V.N. Mai[5,*], Dao Ngoc Nhiem[1,2,*]

[1]Institute of Materials Science, Vietnam Academy of Science and Technology, 18 Hoang Quoc Viet Street, Nghia Do Ward, Hanoi 100000, Vietnam

[2]Graduate University of Science and Technology, 18 Hoang Quoc Viet Street, Nghia Do Ward, Hanoi 100000, Vietnam

[3]Joint Vietnam-Russia Tropical Science and Technology Research Center, 63 Nguyen Van Huyen Street, Nghia Do Ward, Hanoi 100000, Vietnam

[4]Mientrung University of Civil Engineering, 195 Ha Huy Tap Street, Binh Kien Ward, Dak Lak 630000, Vietnam

[5]Faculty of Natural Science, Quy Nhon University, 170 An Duong Vuong Street, Quy Nhon Nam Ward, Gia Lai 610000, Vietnam

**Corresponding author:* nhiemdn@ims.vast.ac.vn and nguyenvungocmai@qnu.edu.vn

***Table S1**. Detailed information of provided activated carbon*

**Activated carbon**	Iodine content	1.041 mg. g^{-1}
	Moisture	2.8%
	Bulk density	506 g. L^{-1}
	pH	6.6
	Grain size	0.425 – 0.85 mm (98.4%)

***Table S2**. LC/MS condition for analysis of PFOS concentration during photocatalysis*

**LC 1290 Infinity - 6430 Triple quad LC/MS Agilent**		
**LC condition**	Column	Zorbax Elcipse plus C18 Rapid resolution (id 2.1×50 mm, grain size: 1.8 μm), Agilent
	Injection volume	3 μL
	Flow rate	0.25 mL/min
	Mobile phase	A: CH_3COONH_4 5 mmol/L<br>B: CH_3OH<br>A:B = 20:80 v/v
**MS condition**	Ion source	MMI
	Scan type	MRM
	Ion mode	ESI
	Precursor ion (m/z)	499
	Product ion (m/z)	80 (for quantification)
	Fragmentor	135 V
	Collision energy	70 V
	Cell Accelerator Voltage	4 V
	Dwell time	200 msec
	Polarity	Negative
	Gas flow	5.1 L. min^{-1}
	Gas temperature	325 ^{o}C
	Nebulizer	60 psi

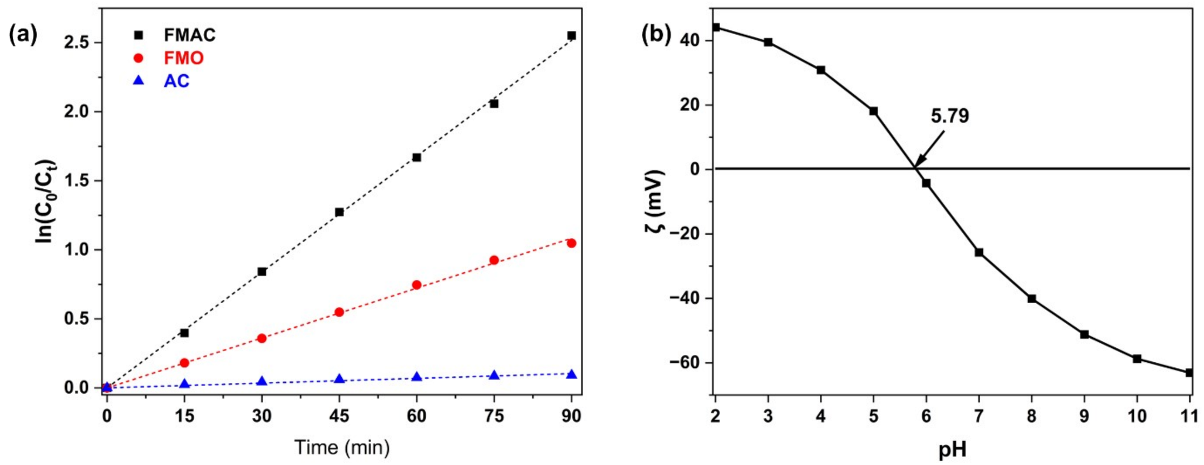

Figure S1. (a) Fitting of first-pseudo-kinetic model for PFOS degradation using different photocatalysts. (b) ζ potential of FMAC sample at different pH

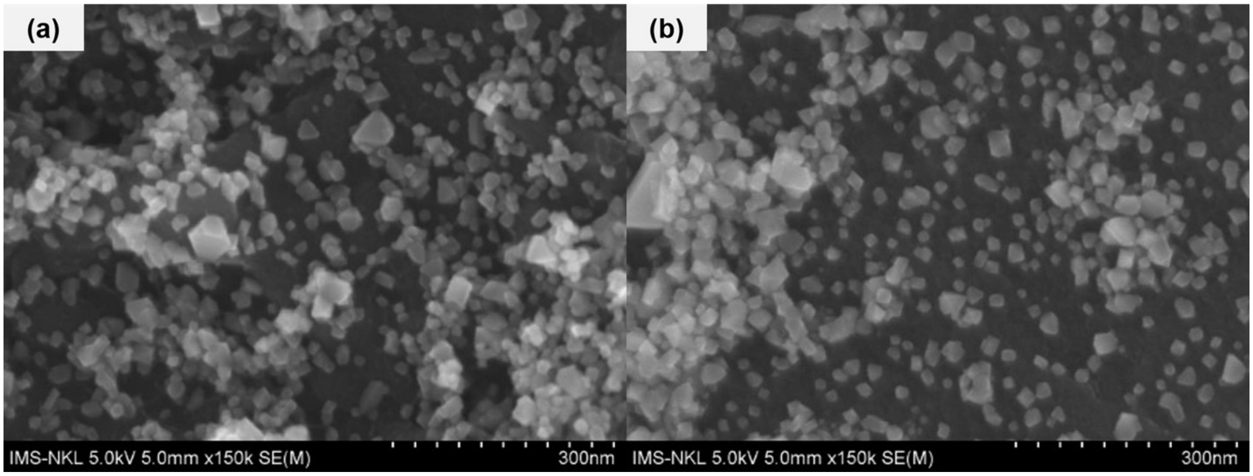

Figure S2. SEM images of FMAC sample (a) before and (b) after durability test for PFOS photodegradation
